# Draft Genome Sequence of a *Salmonella enterica* Serovar Typhi Strain Resistant to Fourth-Generation Cephalosporin and Fluoroquinolone Antibiotics

**DOI:** 10.1128/genomeA.00850-17

**Published:** 2017-10-19

**Authors:** Danish Gul, Robert F. Potter, Hurmat Riaz, Shifa Tariq Ashraf, Meghan A. Wallace, Tehmina Munir, Amjad Ali, Carey-Ann Burnham, Gautam Dantas, Saadia Andleeb

**Affiliations:** a Atta-ur-Rahman School of Applied Biosciences, National University of Sciences and Technology (NUST), Islamabad, Pakistan; b Army Medical College, National University of Medical Sciences, Rawalpindi, Pakistan; c Center for Genome Sciences and Systems Biology, Washington University School of Medicine, St. Louis, Missouri, USA; d Department of Pathology and Immunology, Washington University School of Medicine, St. Louis, Missouri, USA; e Department of Molecular Microbiology, Washington University School of Medicine, St. Louis, Missouri, USA; f Department of Biomedical Engineering, Washington University in St. Louis, St. Louis, Missouri, USA; g Department of Pediatrics, St. Louis Children’s Hospital, St. Louis, Missouri, USA

## Abstract

Typhoid is endemic in developing countries. We report here the first draft genome sequence of a *Salmonella enterica* serovar Typhi clinical isolate from Pakistan exhibiting resistance to cefepime (a fourth-generation cephalosporin) and fluoroquinolone antibiotics, two of the last-generation therapies against this pathogen. The genome is ~4.8 Mb, with two putative plasmids.

## GENOME ANNOUNCEMENT

Typhoid fever is one of the leading causes of mortality due to infectious diseases in developing Asian and African countries. Resistance to third-generation cephalosporin and fluoroquinolone antibiotics has been increasing in recent years in *Salmonella* spp. (1, 2), but there have been few reports of resistance to fourth-generation cephalosporins and fluoroquinolones in *Salmonella enterica* serovar Typhi.

In this study, we performed whole-genome sequencing of a multidrug-resistant *Salmonella enterica* subsp. *enterica* serovar Typhi clinical isolate obtained from a patient admitted to a tertiary care hospital in Rawalpindi, Pakistan (3). The isolate was phenotypically resistant to a fourth-generation cephalosporin (cefepime) and several fluoroquinolones (ciprofloxacin, levofloxacin, and moxifloxacin). Whole-genome sequencing (WGS) was carried out to identify the resistance determinants carried by this *S*. Typhi isolate.

Genomic DNA from 10 colonies of the isolate was extracted with the bacteremia DNA kit (Mo Bio) and used as input for sequencing libraries using a modification of the Nextera XT kit (Illumina) (4). A total of 10,307,350 paired-end 150-bp reads were generated from an Illumina NextSeq 2500 and processed using the High-Throughput Computing Facility at the Washington University School of Medicine (St. Louis, MO, USA). Illumina adapters and potentially contaminating human reads were removed using Trimmomatic and deconseq (5, 6). The resulting 10,288,264 paired-end reads were assembled into scaffolds using SPAdes 3.10.0 (7). Contigs were analyzed by QUAST (8). The assembly produced 95 contigs >500 bp, with an *N*_50_ value of 144,739 bp. The longest contig was 393,347 bp. Contigs were annotated using the NCBI Prokaryotic Genome Annotation Pipeline (PGAP) (http://www.ncbi.nlm.nih.gov/genomes/static/Pipeline.html), which identified 4,975 coding sequences (CDSs), 69 tRNA genes, and 4 rRNA genes.

Further analysis identified putative plasmid sequences and antimicrobial resistance genes. PlasmidFinder 1.3 (https://cge.cbs.dtu.dk/services/PlasmidFinder/) (9) identified the presence of IncQ1 and IncY sequences. Antibiotic resistance genes (ARGs) were identified using ARDB (10), CARD (11), ResFinder (12), and ResFams (13). Annotation using these databases identified *bla*_CTX-M-15_, *bla*_TEM-1_, S83F *gyrA*, and *qnrS1*, which could potentially explain the observed resistance to cefepime and fluoroquinolones (ciprofloxacin, levofloxacin, and moxifloxacin).

The prevalence of *bla*_CTX-M-15_-positive *S*. Typhi isolates of Asian origin has been reported in Iraq, Kuwait, India, and Bangladesh (14–17). To the best of our knowledge, this study is the first draft genome sequence of a *bla*_CTX-M-15_-, *bla*_TEM-1_-, and *qnrS*-positive *S*. Typhi strain from Pakistan exhibiting resistance to cefepime and fluoroquinolones.

### Accession number(s).

This whole-genome shotgun project has been deposited at DDBJ/ENA/GenBank under the accession number NIFP00000000. The version described in this paper is version NIFP01000000.
